# Enhanced Thermoelectric Performance in Cu-Intercalated BiTeI by Compensation Weakening Induced Mobility Improvement

**DOI:** 10.1038/srep14319

**Published:** 2015-09-23

**Authors:** Lihua Wu, Jiong Yang, Miaofang Chi, Shanyu Wang, Ping Wei, Wenqing Zhang, Lidong Chen, Jihui Yang

**Affiliations:** 1 State Key Laboratory of High Performance Ceramics and Superfine Microstructure, Shanghai Institute of Ceramics, Chinese Academy of Sciences, Shanghai 200050, China; 2 Department of Materials Science and Engineering, University of Washington, Seattle, Washington 98195, United States; 3 Materials Genome Institute, Shanghai University, Shanghai 200444, China; 4 Center for Nanophase Materials Sciences, Oak Ridge National Laboratory, Oak Ridge, Tennessee 37831, United States

## Abstract

The low weighted carrier mobility has long been considered to be the key challenge for improvement of thermoelectric (TE) performance in BiTeI. The Rashba-effect-induced two-dimensional density of states in this bulk semiconductor is beneficial for thermopower enhancement, which makes it a prospective compound for TE applications. In this report, we show that intercalation of minor Cu-dopants can substantially alter the equilibria of defect reactions, selectively mediate the donor-acceptor compensation, and tune the defect concentration in the carrier conductive network. Consequently, the potential fluctuations responsible for electron scattering are reduced and the carrier mobility in BiTeI can be enhanced by a factor of two to three between 10 K and 300 K. The carrier concentration can also be optimized by tuning the Te/I composition ratio, leading to higher thermopower in this Rashba system. Cu-intercalation in BiTeI gives rise to higher power factor, slightly lower lattice thermal conductivity, and consequently improved figure of merit. Compared with pristine BiTe_0.98_I_1.02_, the TE performance in Cu_0.05_BiTeI reveals a 150% and 20% enhancement at 300 and 520 K, respectively. These results demonstrate that defect equilibria mediated by selective doping in complex TE and energy materials could be an effective approach to carrier mobility and performance optimization.

Carrier mobility in materials plays an important role in energy storage and conversion, as epitomized in batteries, photovoltaics, and thermoelectrics^1^,^2^,^3^,^4^. In lithium-ion battery cathodes, the electron mobility needs to be high enough to match the diffusion speed of lithium ions^1^. As for the thin-film solar cells, sufficient electron mobility is favored for the transparent conductive oxide, while the photovoltaically active layer should possess high carrier mobility × lifetime products for electrons and holes^2^,^5^. Meanwhile, the electrical performance of thermoelectric (TE) materials fundamentally relies on the carrier mobility *μ*_*H*_ and more specifically on the weighted mobility *μ*_*H*_ (m^*^/m_*e*_)^3/2^, where m^*^ and *m*_*e*_ are the carrier effective mass and free electron mass, respectively^6^,^7^. Improving the carrier mobility is challenging but significant for all high-performance energy materials. In principle, carriers cannot “see” the periodically-arranged ions in perfect crystals, as no collisions occur for carriers in the periodic potential. However, perturbations from lattice imperfections, impurities, and thermal vibrations of the ions can scatter the carriers and deteriorate the carrier transport mobility in materials^8^.

TE technology is one potential candidate which can facilitate direct thermal-to-electrical energy conversion^9^. TE materials, combinations of fine electrical and poor thermal conductors, are the keys to improving the efficiency of this green technology. The performance of a TE material is determined by its dimensionless figure of merit 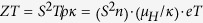, where *S* is the thermopower, *T* the absolute temperature, *ρ* the electrical resistivity, *κ* the thermal conductivity, *n* the carrier concentration, and *e* the electron charge^10^,^11^. Doping strategies and defect related approaches have been successfully used to optimize TE properties in several classes of high efficiency TE material systems, such as CoSb_3_^12^,^13^,^14^, Mg_2_(Si, Sn)^15^, Bi_2_Te_3_^16^, and Pb(Se, Te)^17^,^18^. Narrow gap semiconductors with low iconicity generally should have high carrier mobility^19^. However, methods to optimize the TE performance, such as doping to tune the carrier concentration and alloying to engineer the band structure or reduce lattice thermal conductivity, would inevitably introduced disorders and randomness to the materials, Thus, the carrier mobility *μ*_*H*_ can be deteriorated to unexpectedly low values^6^.

In intrinsic semiconductors, the predominant carrier scattering source is the acoustic phonons, as shown in most conventional TE materials^20^. The mobility is coupled with carrier concentration (the Fermi level) by the deformation potential^21^. This situation leaves little room for mobility tuning. On the other hand, many complex TE compounds, including ternary and multinary materials, belong to the family of compensated semiconductors in the extrinsic regime, where all impurities are ionized, the transport carriers are generated by but at the same time strongly scattered by these ionized impurities including both acceptors and donors^22^. These localized scattering centers break the lattice periodicity and create electrostatic potential fluctuations. In real crystal lattices, the defects and imperfections in carrier conductive network can cause the undesirable potential fluctuations and strongly scatter the carriers. Carrier mobility is closely related to the concentration of ionized impurities *N*_*I*_, *N*_*I*_ = *N*_*A*_ + *N*_*D*_, where *N*_*A*_, *N*_*D*_ are the concentration of negative-charged acceptors and positive-charged donors, respectively. Taking an *n*-type extrinsic semiconductor as an example, the electron concentration *n* can be expressed as *n* = *N*_*D*_ − *N*_*A*_^23^. Then at a given electron concentration *n* (the optimal *n* is 10^19^–10^20^ cm^−3^ for most TE materials), the impurity concentration is primarily determined by the compensation ratio *K* = *N*_*A*_*/N*_*D*_. A Monte Carlo study clearly shows that compared with the situation in single-type-charged impurity (*K* = 0), impurities of two different charges (0 < *K* < 1) can lead to more severe potential fluctuations around the mean potential value^24^. Larger compensation ratio *K* will result in stronger potential fluctuations, which can strongly scatter transport carriers and thus lower the carrier mobility^24^,^25^. This study also implies that reducing *K* may become an unusual avenue to increase the carrier mobility and enhance the TE performance in systems with high compensation.

Until recently, non-centrosymmetric Rashba spin-split bismuth tellurohalides BiTe*X* (*X* = I, Br, Cl) have been reported as promising TE materials^26^,^27^,^28^,^29^,^30^. The electrical performance of BiTeI is superior than those in spin-degenerate materials, while the lattice thermal conductivity of undoped polycrystalline BiTeI is as low as ~1 W m^−1^ K^−1^ at room temperature^29^. However, the overall TE performance of BiTeI is still unassuming because of its low weighted mobility^6^. Antisite defects occur in BiTeI crystals owing to the similarity between Te and I atoms. Thus the dominant defects can be expressed as 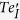 and 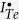. High concentrations of ionized impurities, including acceptors and donors, exist in this layered material^31^. Consequently, the electrons are scattered by both the ionized impurities and the acoustic phonons^31^,^32^. This material can be an ideal testbed for tuning carrier mobility by altering the impurity compensation.

Low temperature TE characterizations of BiTeI single crystals were performed in the 1970s^32^. A recent resurgence of interests in the TE properties of BiTeI included band structure calculations and galvanomagnetic characterizations^27^. It was found that thermopower of BiTeI can be enhanced with small amounts of CuI additions, while both thermal conductivity and electrical conductivity decrease in polycrystalline samples^26^,^33^. The TE figure of merit nevertheless deteriorates as compared with the undoped single crystals. It was also speculated that the Bi^3+^ cations were substituted by the Cu^+^ cations, thus reducing the carrier concentration. More recently, magneto-transport measurements have been performed on BiTeI single crystals with much higher Cu contents, where copper ions were argued to distribute randomly within the van der Waals gaps^34^. Cu-doping was shown to reduce the carrier concentration and increase the mobility. The occupancy of Cu in BiTeI and its influence on the transport properties are important for both spintronics and thermoelectrics.

Here we present that the intercalation of minor Cu dopants in BiTeI polycrystals can clearly change the equilibria of defect reactions, selectively mediate the compensation between donors and acceptors, and limit the defect concentration in the carrier conductive network. The intercalated 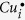 donors alter the compensation ratio between the donors (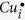 and 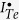) and the acceptors (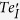). Strikingly, the electron mobility is enhanced by three and two times at 10 K and 300 K, respectively. The significantly enhanced carrier mobility is driven by the weakened impurity compensation and reduced potential fluctuations. The carrier concentration of BiTeI is also optimized by Cu-intercalation and tuning the Te/I composition ratio, beneficial to power factor (*PF* = *S*^2^/*ρ*) enlargement. Cu intercalation induced *ZT* improvement for BiTeI is 150% and 20% at 300 K and 520 K, respectively, which mainly originates from the mobility enhancement and the carrier concentration optimization.

## Results and Discussion

**Figure 1:**
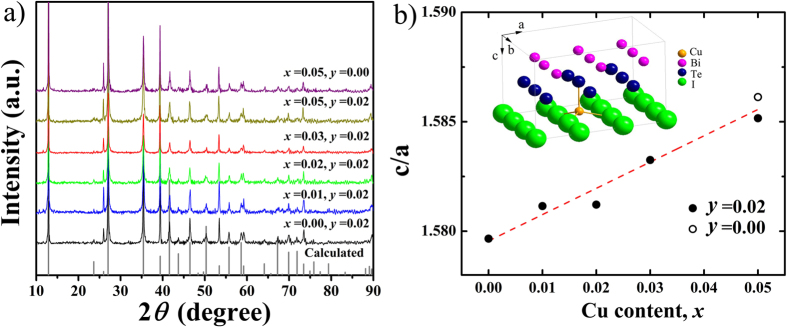
(**a**) XRD patterns and (**b**) ratios of lattice parameters (c/a) for all Cu_*x*_BiTe_1−*y*_I_1+*y*_ samples. The dashed line in (**b**) is a guide for the eye. The inset of (**b**) shows the crystal structure of BiTeI, where the intercalated Cu atom locates in the van der Waals gap.

Figure 1(a) shows the powder X-ray diffraction (XRD) patterns of all Cu_*x*_BiTe_1−*y*_I_1+*y*_ samples with the nominal compositions *x* = 0.00, 0.01, 0.02, 0.03, 0.05, *y* = 0.02; and *x* = 0.05, *y* = 0.00, confirming the phase purity. Compared to the calculated diffraction peaks of BiTeI, these samples show a preferred orientation of (0 0 1). Lattice parameter ratios (c/a) of the samples (Fig. 1(b)) are calculated from cell refinements using the space group P3*m*1 and experimental lattice parameters a = b = 4.3392 Å and c = 6.8540 Å^35^. The pseudo-binary phase diagram between Bi_2_Te_3_ and BiI_3_ shows that stoichiometric BiTeI does not exist^36^. We also find that the Bi_2_Te_3_ secondary phase is always present in the stoichiometric BiTeI, while the non-stoichiometric BiTe_0.98_I_1.02_ is phase pure. However, single phase Cu_0.05_BiTeI can be formed without any impurities according to the XRD pattern.

In general, the lattice site occupancies of dopants have a significant impact on physical properties. There are two potential sites for Cu dopants in the layered BiTeI, i.e. substitution on the Bi site and intercalation into the van der Waals gap^26^,^34^. The c/a ratio increases with increasing Cu content *x*, as shown in Fig. 1(b), which suggests that intercalation is more likely. Formation energy calculations were performed to further determine the preferential site for Cu atoms^37^,^38^. In a Bi_27_Te_27_I_27_ supercell, the defect reactions for substitution and intercalation can be expressed as


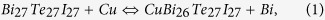



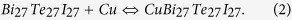


The formation energy for Cu-substitution on the Bi site and Cu-intercalation is calculated to be 2.24 eV and 1.17 eV, respectively. The intercalation requires lower energy than the substitution by 1.07 eV, further corroborating Cu-intercalation in BiTeI. The intercalated Cu atoms are located in the tetrahedrons formed by three I atoms and one Te atom, as shown in the inset of Fig. 1(b). The length of Cu-I and Cu-Te bond is calculated to be 2.561 Å and 2.502 Å, respectively.

**Figure 2:**
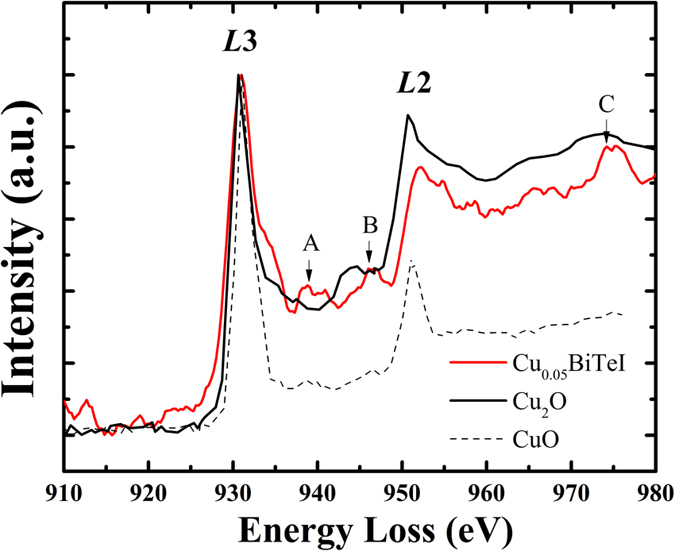
Typical EELS plot of the Cu-*L*3 and *L*2 edges for Cu_0.05_BiTeI sample, compared with spectra of standard Cu_2_O and CuO references.

The electron energy loss spectrum (EELS) was used to measure the oxidation state of Cu in Cu_0.05_BiTeI by analyzing its *L*3 and *L*2 fine structures, as shown in Fig. 2. The acquired spectrum was compared to those from standard Cu_2_O and CuO^39^. The spectra are aligned to the *L*3 peak intensity maxima in order to have a clear comparison of the fine structures and peak intensity ratio. The fine structures of *L*3, *L*2 edges and the *L*3-to-*L*2 ratio on the EELS, can be used as fingerprints of the oxidation states for transition metal elements^40^,^41^. The spectrum for the Cu-intercalated BiTeI sample has the *L*3 and *L*2 sharp edges and intense peaks, which excludes the zero-valence state of Cu^42^. The spectrum in this work sits between those from Cu_2_O and CuO in terms of the fine structures. For example, the *L*2 peak intensity from Cu_0.05_BiTeI is lower than that of Cu_2_O but higher than that of CuO. The fine structures between *L*3 and *L*2, which are labeled as peak “A” and “B”, resemble those of CuO, while the extended fine structure shows an intense peak (labeled as peak “C”), which is similar to that of Cu_2_O. Furthermore, the *L*3-to-*L*2 ratio of Cu_0.05_BiTeI is also between those in standard Cu_2_O and CuO. These evidences indicate that the Cu oxidation state in Cu-intercalated BiTeI is between 1+ and 2+. The Cu ionization has a significant influence on the transport properties of these samples.

**Figure 3:**
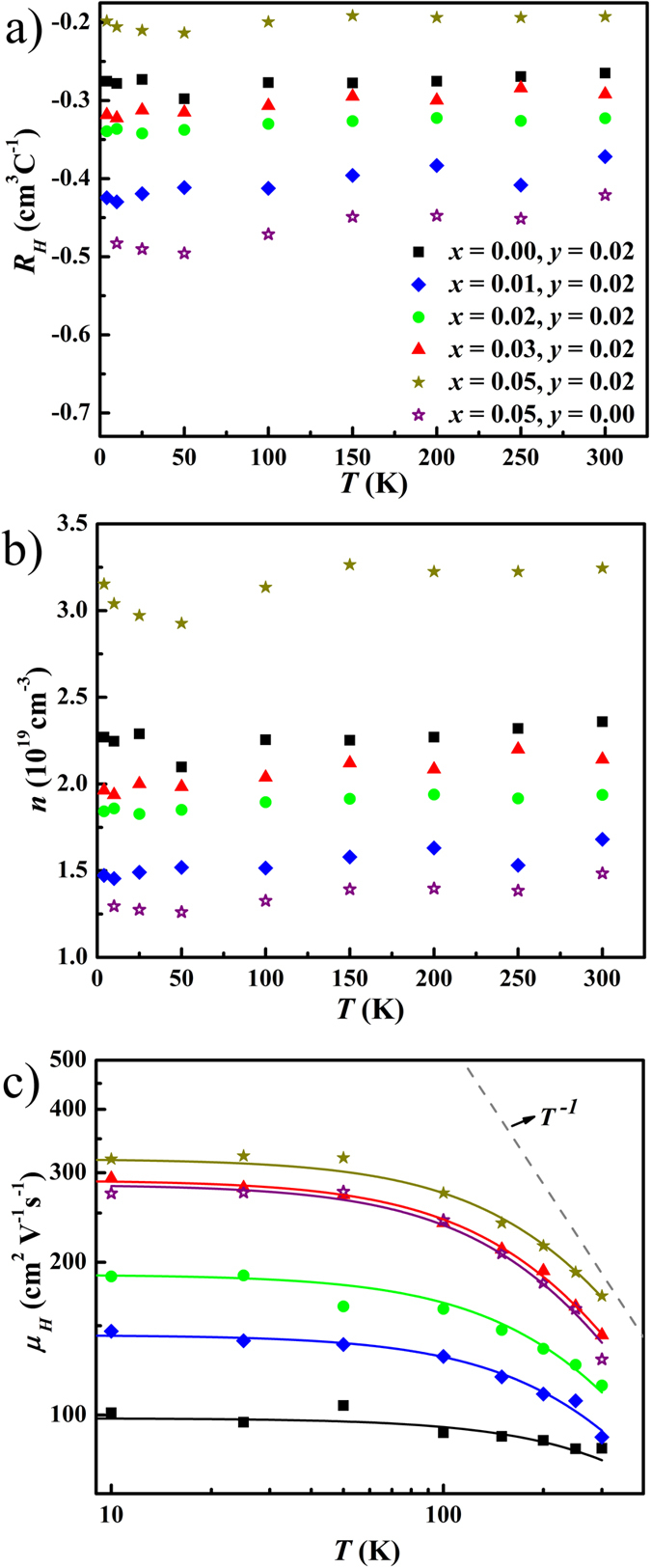
Temperature dependence of (a) Hall coefficient *R*_*H*_, (b) carrier concentration *n*, and (c) mobility *μ*_*H*_ for Cu_*x*_BiTe_1−*y*_I_1+*y*_ samples. Solid lines in (**c**) are fitted using a mixed scattering model, while the dashed line represents a *μ*_*H*_ ∝ *T*^ −1^ relationship.

Figure 3(a) shows that all Cu_*x*_BiTe_1–*y*_I_1+*y*_ samples have negative Hall coefficients *R*_*H*_, suggesting *n*-type electron-dominant conduction. The carrier concentration *n* is nearly temperature independent (Fig. 3(b)), which indicates that these samples are in the extrinsic region where all donors and accepters are ionized^22^. The carrier concentration shows a non-monotonic dependence on the copper content *x* in the Cu_*x*_BiTe_0.98_I_1.02_ samples. The room temperature carrier concentration decreases from 2.35 × 10^19^ to 1.68 × 10^19^ cm^−3^, when *x* increases from 0.00 to 0.01, and then increases to 3.24 × 10^19^ cm^−3^ for the *x* = 0.05, *y* = 0.02 sample. The Cu_0.05_BiTeI (*y* = 0) sample has the lowest carrier concentration.

Most strikingly, the Hall mobility *μ*_*H*_ is significantly enhanced by Cu intercalation, as shown in Fig. 3(c). The Hall mobility of the undoped sample is 92.3 cm^2^ V^−1^ s^−1^ at 100 K and 86.0 cm^2^ V^−1^ s^−1^ at 300 K, while the Cu_0.05_BiTe_0.98_I_1.02_ sample has a mobility as high as 273.6 and 171.6 cm^2^ V^−1^ s^−1^ at the respective temperatures. The mobility of some BiTeI single crystals exhibits a *T*^−3/2^ dependence, which suggests that carriers are predominantly scattered by the acoustic phonons^36^. Meanwhile, the mobility in other BiTeI single crystals displays a weaker temperature dependence^31^,^32^. The temperature dependence of mobility for these BiTeI single crystals has been discussed which includes ionized impurity and acoustic phonon scatterings^31^,^32^. Our mobility data for polycrystals are consistent with the mixed scattering model in the range of 4–300 K, which have the temperature dependence weaker than the *T*^−1^ relationship. If carriers are scattered by grain barriers, such as grain boundary phases or oxidized phases, the mobility would increase with increasing temperature^43^. Our mobility data for polycrystalline BiTeI show no such temperature dependence, which excludes such extrinsic scattering factors.

Therefore, the Hall mobility *μ*_*H*_ can be analyzed by combining the scattering processes using the Matthiessen’s law^44^


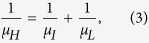


where *μ*_*I*_ and *μ*_*L*_ represent the mobility component related to the ionized impurity and the acoustic phonon scattering, respectively. The acoustic phonon limited mobility *μ*_*L*_ can be described by the equation^21^


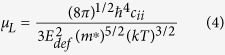


or


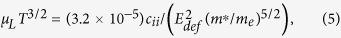


where *c*_*ii*_ and *E*_*de*f_ represent the average elastic constant (in the centimeter-gram-second unit of Ba) and the deformation potential (in the unit of eV), respectively. The other constants include the electron mass *m*_*e*_, the reduced Planck constant 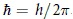, and the Boltzmann constant *k*. The effective mass m* of BiTeI is about 0.19 *m*_*e*_ determined by the Shubnikov-de Haas oscillations^45^. The average elastic constant *c*_*ii*_ of BiTeI is estimated to be 1.36 × 10^11^ Ba by using the relation 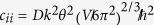, where *D* is the density, *θ* the Debye temperature, and *V* the unit cell volume^46^. The Debye temperature *θ* is calculated to be 87 K by taking the equation 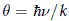, where *𝜈* is the maximum frequency of acoustic branches (61 cm^−1^) given by the calculated phonon dispersion (shown below).

The mobility component *μ*_*I*_ associated with the ionized impurity scattering can be expressed by the Brooks-Herring (BH) formula in the limit of electron degeneracy^47^,^48^


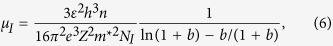


where 
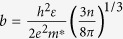
. In the BH formula, *ε* is the dielectric constant and equals to 14.5 for BiTeI^49^. The parameters *N*_*I*_ and *Z* refer to the concentration of ionized impurity and charge of impurity (*Z* = 1).

Using Equations 3, 5 and 6, we can fit the experimental Hall mobility data with two variables, i.e. deformation potential *E*_*de*f_ and concentration of ionized impurity *N*_*I*_. The solid lines in Fig. 3(c) present the fits to mobility data and Table 1 lists the fitting parameters. The deformation potential *E*_*de*f_ (determined by the fits) is 10.6 eV for the undoped BiTe_0.98_I_1.02_ sample, and 12.1–14.2 eV for Cu-doped samples. The undoped sample has very high impurity concentration (*N*_*I*_ = 18.1 × 10^19^ cm^−3^). Cu-intercalation significantly reduces the impurity concentration, all Cu-intercalated samples have impurity concentrations *N*_*I*_ lower than 10.0 × 10^19^ cm^−3^. In this extrinsic region, the concentrations of donors (*N*_*D*_) and acceptors (*N*_*A*_), as well as the compensation ratio (*K*) can be calculated by using the relations *n* = *N*_*D*_ − *N*_*A*_ and *N*_*I*_ = *n* + 2*N*_*A*_^23^. The calculated *N*_*D*_, *N*_*A*_ and *K* are also listed in Table 1. Donor and acceptor concentrations both decrease drastically upon Cu-intercalation. The compensation ratios become smaller in Cu-intercalated samples, as compared with that in the undoped BiTe_0.98_I_1.02_ (*K* = 76.9%). The strong compensation in BiTeI is closely related to the similarity between Te and I atoms, including the ionic radii and atomic charges^50^. The two atoms can exchange their positions randomly in the crystal structure^31^, forming the compensated antisite defects. The antisite defect reaction in the strongly-compensated BiTeI can be simplified as


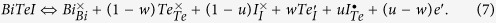


The coefficients in Equation 7 have the condition of *u* > *w*, which is the origin of the *n*-type conductio*n* in BiTeI.

**Figure 4:**
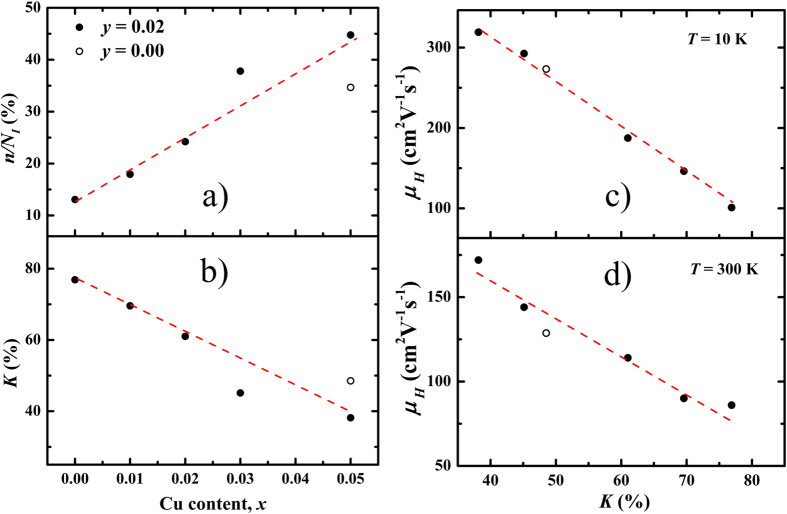
Copper content *x* dependent (a) ratio between carrier *n* and impurity concentrations *N*_*I*_, and (b) compensation ratio *K* for Cu_*x*_BiTe_1−*y*_I_1+*y*_. Compensation ratio *K* dependent mobility at (**c**) 10 K and (**d**) 300 K. The dashed lines are guides for the eye.

**Figure 5:**
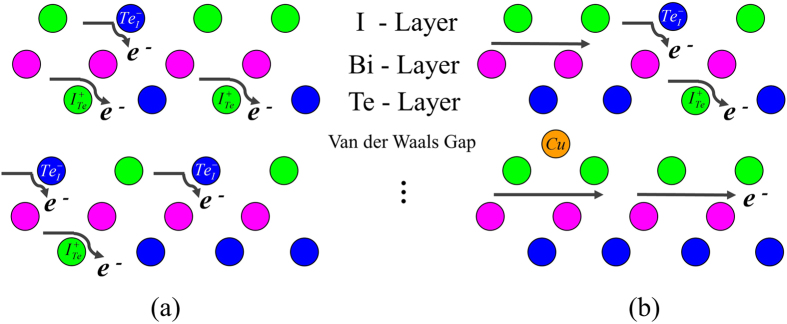
Schematic diagrams of the balance of defect equilibria and electron scattering in BiTeI, (a) before and (b) after Cu-intercalation. In pristine BiTeI, highly compensated antisite defects can generate large potential fluctuations, which strongly scatter electrons and lead to low carrier mobility. Upon Cu-intercalation, the intrinsic defect equilibrium is altered and the compensation ratio is reduced. Electron scattering from the compensated defects is weakened. Consequently, higher carrier mobility can be achieved in Cu-intercalated BiTeI.

Figure 4 sheds light on the compensation-related physical parameters and their interrelations to the Hall mobility *μ*_*H*_. The Cu content *x* dependent *n/N*_*I*_ and *K* are shown in Fig. 4(a,b). The term *n/N*_*I*_ describes the “efficiency” of ionized impurities for providing the conducting carriers. As *x* increses from 0.00 to 0.05 (*y* = 0.02), the efficiency *n/N*_*I*_ increases from 13.1% to 44.8%. Meanwhile, the compensation ratio *K* decreases from 76.9% to 38.2%. For the *x* = 0.05, *y* = 0.00 sample, the values of *n/N*_*I*_ and *K* are close to that of the *x* = 0.03, *y* = 0.02 sample. Therefore, the ionizations are more efficient with weaker compensation and less impurities in the Cu-intercalated samples. Figure 4(c,d) present the compensation ratio *K* dependent Hall mobility *μ*_*H*_ at 10 K and 300 K. At 10 K, *μ*_*H*_ increases almost linearly with the decreasing *K*, consistent with the picture that the mobility is mainly determined by the ionized impurity scattering at lower temperatures (below 100 K). Although acoustic phonon scattering emerges at higher temperatures, the Hall mobility is still strongly influenced by the impurity scattering in the compensated BiTeI. As shown in Fig. 4(d), the Hall mobility *μ*_*H*_ at 300 K also varies inversely with the compensation ratio *K*. Based on the EELS results, the defect reaction of Cu intercalation in Equation 2 can be simplified as (taking the oxidation state 1+ of Cu as an example)


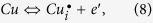


which provides electrons and thus increases the electron concentration. From the Le Chatelier’s principle^51^, the position of equilibrium in Equation 7 moves to the left side for mitigating the electron concentration increase as the Cu content increases. Consequently, the concentrations of intrinsic antisite defects (donors and acceptors) also decrease, as schematically shown in Fig. 5. The reaction in Equation 8 has no compensation effect, as intercalated Cu only act as donors. The overall compensation ratio *K* is then suppressed by the ionization of intercalated Cu. The compensation ratio *K* in particular captures the electrostatic potential fluctuations in semiconductors. If there are only negatively charged acceptors or positively charged donors in materials, *K* equals to zero. The potential fluctuations with respect to their mean potential value become larger with increasing compensation ratio *K*. Larger potential fluctuations cause stronger scattering for the electrons, thus reducing the carrier mobility^24^. In BiTeI, Cu-intercalation reduces the compensation ratio, i.e. the potential fluctuations, and weakens the electron scattering by ionized impurities, as shown in Fig. 5(b). Hence the mobility can be significantly enhanced in Cu-intercalated BiTeI. Actually, the enhanced mobility here can also be described as increased weighted mobility, as the effective mass should not altered by minor Cu dopants. The mobility enhancement has also been found in Cu-intercalated layered Bi_2_Te_3_^52^,^53^, which may have the same underlying mechanism. In general, dopants in semiconductors, that occupy lattice sites with minimal influence on the charge-conducting band^54^ or carrier-conduction network^55^, could lead to higher carrier mobility. The intercalated Cu atoms reduce the perturbation in the carrier-conducting Bi-Te network. Herein we only considered the long-range interaction in ionized impurity scattering, due to the relatively low dielectric constant (14.5) in BiTeI. However, short-range interactions may be dominant in compounds with large dielectric constant, which can also be tuned for higher mobility^54^.

**Figure 6:**
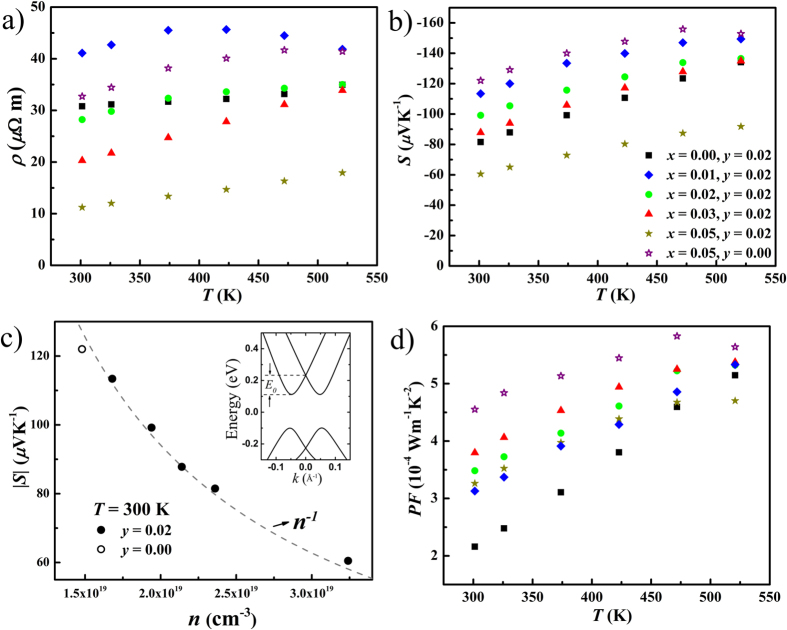
Temperature dependence of (a) resistivity *ρ* and (b) thermopower *S* for Cu_*x*_BiTe_1−*y*_I_1+*y*_. (**c**) Relation between thermopower *S* and carrier concentration *n* at 300 K. The inset in (**c**) is the calculated band structure of BiTeI, while the dashed line shows a *S* ∝ *n*^−1^ relationship. (**d**) Temperature dependent power factor (*PF*) for Cu_*x*_BiTe_1−*y*_I_1+*y*_.

Figure 6(a,b) show the electrical resistivity *ρ* and thermopower *S* of Cu_*x*_BiTe_1−*y*_I_1+*y*_ as a function of temperature (300–520 K). These samples exhibit a metallic behavior in resistivity, which is typical for degenerate semiconductors. The non-systematic resistivity variation with copper doping has been revealed in the carrier concentration and mobility. Compared to the doped samples, the resistivity of pure BiTe_0.98_I_1.02_ (*x* = 0.00, *y* = 0.02) demonstrates a weaker temperature dependence, which is compatible with its temperature-dependent Hall mobility. For Cu_0.01_BiTe_0.98_I_1.02_ (*x* = 0.01, *y* = 0.02) and Cu_0.05_BiTeI (*x* = 0.05, *y* = 0.00), the resistivity starts decreasing as holes are excited at higher temperatures.

The negative thermopower *S* of all samples is consistent with the Hall coefficient *R*_*H*_, confirming the dominant *n*-type conduction. All samples show degenerate behaviors near room temperature. Unlike other samples, Cu_0.01_BiTe_0.98_I_1.02_ and Cu_0.05_BiTeI have the bipolar effect at higher temperatures as holes are excited. The variation of room temperature thermopower *S* can be related to the carrier concentration *n*, as shown in Fig. 6(c). The absolute value of *S* increases with decreasing *n*, showing an *S* ∝ *n*^−1^ dependence. The spin-splitting bands have a two-dimensional-like constant density of states^56^,^57^, that can have a significant influence on the TE properties of the Rashba materials^58^, The spin-splitting bands of BiTeI have been revealed by an angle-resolved photo-emission spectroscopy (ARPES) experiment and first principles calculations^50^,^59^. We’ve demonstrated that the electrical term *S*^*2*^*n* can indeed be enhanced because of the unusual density of states in the Rashba bands^29^,^30^. Our work also showed that the lower Fermi level for a given optimal carrier concentration is the underlying mechanism for electrical property enhancement^29^,^30^. In conventional semiconductors with parabolic bands, the *S*-*n* plot typically follows an *S* ∝ *n*^−2/3^ relationship. We have previously developed a theoretical model to address the thermopower vs. the carrier concentration relationship in the bulk Rashba system^29^. Under a degenerate approximation, the thermopower in bulk materials with Rashba spin-splitting bands can be written as


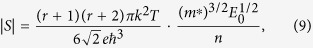


where *E*_0_ is the Rashba energy and *r* the carrier scattering parameter. The band structure of BiTeI has been calculated (inset of Fig. 6(c)), showing the Rashba energy *E*_0_ = 0.11 eV, which is comparable to the ARPES and previous calculation results^50^,^59^. The scattering parameter *r* is −0.5 and 1.5 for the acoustic phonon and the ionized impurity scattering, respectively^20^. The dashed line in Fig. 6(c) corresponds to calculated results using Equation 9 with the scattering parameter *r* equal to *−*0.096, indicative of the mixed scatterings in BiTeI, which is consistent with our Hall mobility analysis. The temperature dependent power factor *PF* = *S*^*2*^*/ρ* is shown in Fig. 6(d). The significant *PF* enhancement in Cu-doped samples is attributed to the improvement of electrical term *S*^*2*^*n* and mobility *μ*_*H*_. The *PF* in the Cu_0.05_BiTeI (*x* = 0.05, *y* = 0.00) sample is 2.3 times higher than that of the undoped sample at room temperature.

**Figure 7:**
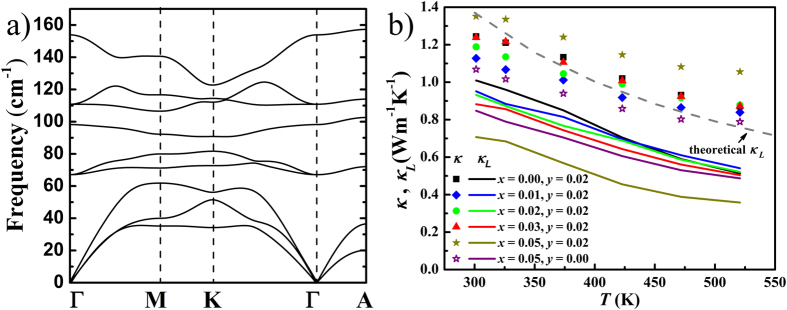
(a) Calculated phonon dispersion of BiTeI. (**b**) Temperature dependence of total thermal conductivity *κ* (dots) and lattice thermal conductivity *κ*_*L*_ (solid lines) for Cu_*x*_BiTe_1−*y*_I_1+*y*_. The dashed line in (b) represents theoretical lattice thermal conductivity.

Figure 7(a) shows the calculated phonon dispersion of BiTeI along the high symmetry points in the reciprocal space. The maximum frequency is 157 cm^−1^ for all phonon branches and 61 cm^−1^ for the acoustic branches. Three heat-carrying acoustic phonon branches are related to the lattice vibrations of all atoms^60^. Figure 7(b) shows the temperature dependent thermal conductivity of the Cu_*x*_BiTe_1−*y*_I_1+*y*_ samples with different values of *x* and *y*. The thermal conductivity of all samples decreases with increasing temperature, and is lower than 1 W m^−1^ K^−1^ at 520 K for most samples. The total thermal conductivity can be divided to two components by *κ* = *κ*_*e*_ + *κ*_*L*_, where *κ*_*e*_ and *κ*_*L*_ represent the electronic and lattice thermal conductivity, respectively. *κ*_*L*_ can be calculated by deducting *κ*_*e*_ from *κ* using the Wiedemann-Franz law *κ*_*e*_=*LT*/*ρ*, where *L* is the Lorenz number and equals to 2.45 × 10^−8^ V^2^ K^−2^. The choice of the Lorenz number here does not alter the overall conclusion we present. As the solid lines in Fig. 7(b) show, the derived *κ*_*L*_ of Cu_*x*_BiTe_0.98_I_1.02_ decreases as the copper content increases from *x* = 0.00 to *x* = 0.05. The Cu_0.05_BiTe_0.98_I_1.02_ sample has *κ*_*L*_ as low as 0.36 W m^−1^ K^−1^ at 520 K, which is close to the theoretical minimum *κ*_*L*_ of 0.3 W m^−1^ K^−1^ 61. The lattice thermal conductivity can be calculated via the Debye-Callaway method^62^, as the dashed line shows in Fig. 7(b). The temperature dependence of theoretical values is reasonably consistent with the experimental results of undoped sample. Above room temperature, the *κ*_*L*_ of all samples follows the *T*^−1^ relationship, proving that the phonon-phonon interaction is dominant in the temperature range. The lower *κ*_*L*_ in Cu-intercalated samples is a result of the additional phonon scattering by point defects, especially the light Cu breaks the periodicity of the layered structure and introduces large mass fluctuations in BiTeI.

**Figure 8:**
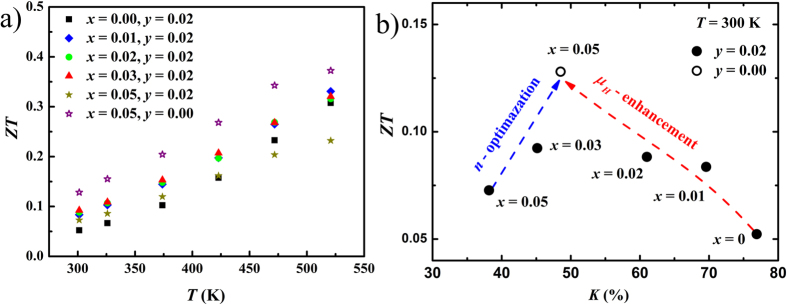
(**a**) Temperature and (**b**) compensation ratio dependent figure of merit *ZT* for all Cu_*x*_BiTe_1−*y*_I_1+*y*_ samples.

Figure 8(a) shows the overall figure of merit *ZT* of all Cu_*x*_BiTe_1−*y*_I_1+*y*_ samples. The *ZT* for Cu_0.05_BiTeI has a ~150% and 20% enhancement at 300 K and 520 K over the undoped sample, respectively. The *ZT* enhancement in Cu-intercalated BiTeI is a joint result of power factor improvement and thermal conductivity reduction. In fact, the mobility enlargement and carrier concentration optimization are the main reasons for the enhanced *ZT* in Cu_0.05_BiTeI, as shown in Fig. 8(b). The high temperature TE property of these materials is, however, still limited by the bipolar effect in the best sample, whose thermopower is suppressed above 475 K. Through substituting iodine with bromine, the band gap of the solid solution may become larger than the pristine BiTeI to mitigate the bipolar conduction at high temperatures^63^.

## Conclusions

In conclusion, we have demonstrated that mobility enhancement via compensation weakening is an effective method for improving the TE properties in BiTeI. Cu-intercalation changes the balance of defect equilibria and weakens the compensation between donors and acceptors. As a result, the potential fluctuations are reduced in the carrier conductive network and electron scattering are substantially suppressed, leading to enhanced electron mobility. The carrier concentration is also tuned by Cu-intercalation and higher thermopower is shown in this Rashba spin-splitting system. An enhanced power factor and lowered lattice thermal conductivity yield higher figure of merit *ZT* in Cu-intercalated BiTeI. It is further suggested that even better performance can be achieved in BiTeI. The beneficial effects of defect equilibria mediation and compensation weakening we demonstrated in BiTeI should be applicable to other complex TE and energy materials.

## Methods

Polycrystalline Cu_x_BiTe_1−y_I_1+y_ samples were synthesized by melting stoichiometric elements of Bi, Te, I_2_, and Cu (99.999%, Sigma-Aldrich) in sealed quartz tubes. The mixtures were first kept at 135 °C (above the melting point of iodine) for 2 h for pre-reaction, and then held at 720 °C for 24 h. Melts were subsequently slow-cooled to 510 °C and held for three days before cooled in furnace. The grown ingots were ground into fine powders, which could be filtered by a 75 μm standard sieve. Powders (weighted around 10 g) were sintered to consolidated bulk samples at 370 °C in a Spark Plasma System (SPS) using a graphite die with a diameter of 12.7 mm under a pressure of 40 MPa.

Powder X-ray diffraction (XRD) was performed at room temperature on a Bruke Focus D8 equipped with Cu K*α* radiation (wavelength *λ* = 1.5418 Å). EDS and elemental mapping was carried out on a Hitachi TM 3100. The EELS spectrum from Cu_0.05_BiTeI is acquired on a FEI-Titan 300/60S STEM/TEM at 300 kV using a Gatan GIF 865 spectrometer. The beam convergence angle and collection angle used in this experiment are 30 mrad and 45 mrad, respectively. The spectrum is averaged on 20 individual acquisitions. The thermopower (*S*) and electrical resistivity (*ρ*) from 300 K to 573 K were measured on an ULVAC ZEM-3 system using bar samples (2.5 × 2.5 × 10 mm^3^), which were cut from sintered materials vertically to the direction of SPS pressure. Thermal conductivity was determined by thermal diffusivity (*α*), density (*D*) and heat capacity (*C*_p_) using the equation *κ* = *C*_p_ × *D* × *α*. Thermal diffusivity (*α*) was measured by a Netzsch LFA 457, vertically to the direction of SPS pressure on square samples with dimensions of ~10 × 10 × 1.5 mm^3^. Archimedes method was used for density of sintered samples while heat capacity was estimated by the Dulong-Petit law. Estimated uncertainties of the measured electric resistivity, Seebeck coefficient, and calculated thermal conductivity are ±5%, ±3%, and ±10%, respectively^19^,^64^,^65^,^66^. Hall measurements were realized on thin bar samples (2 × 9 × 0.6 mm^3^) in a Janis cryostat equipped with an AC resistance bridge and a 9-T magnet (up to ±4 *T* used in this work). The currents (±2 *mA*) for all Hall measurements were applied perpendicular to direction of the SPS pressure while magnetic fields were in parallel. Hall coefficient (*R*_H_) was calculated from slope of Hall voltage *vs.* magnetic field curves. Carrier concentration (*n*) and Hall mobility (*μ*_H_) were calculated from measured Hall coefficients and resistivity using 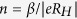 and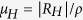, respectively, where *β* approximately equals to one in the degenerate BiTeI.

Defect formation energies are calculated in a Bi_27_Te_27_I_27_ supercell, with the Cu atom in the substitution site or the intercalation site. The change of Fermi energy (~0.026 eV) is small compared to the defect formation energy (2.24 eV and 1.17 eV). Thus the values of formation energy correspond to the proper electron chemical potentials. Band structure of BiTeI was calculated by the Density Functional Theory, where the modified Becke-Johnson (mBJ) exchange potential and the local density approximation (LDA) were used. The spin-orbit coupling was fully taken into account in the calculations. In theoretical lattice thermal conductivity calculation, the calculated phonon velocities are 1543, 1849, and 2316 m s^−1^ for three acoustic branches, while the average Grüneisen parameters are 2.42, 1.76, and 2.10, respectively.

## Additional Information

**How to cite this article**: Wu, L. *et al.* Enhanced Thermoelectric Performance in Cu-Intercalated BiTeI by Compensation Weakening Induced Mobility Improvement. *Sci. Rep.* **5**, 14319; doi: 10.1038/srep14319 (2015).

## Figures and Tables

**Table 1:** Mobility fitting parameters for all samples with different nominal composition, including deformation potential *E*_*de*f_ and concentration of ionized impurity *N*_*I*_.

Nominal composition	*E*_*def*_ [eV]	*N*_*I*_ [10^19^ cm^−3^]	*N*_*D*_ [10^19^ cm^−3^]	*N*_*A*_ [10^19^ cm^−3^]	*K* [%]
BiTe_0.98_I_1.02_	10.6	18.1	10.2	7.85	76.9
Cu_0.01_BiTe_0.98_I_1.02_	14.2	9.37	5.52	3.84	69.6
Cu_0.02_BiTe_0.98_I_1.02_	14.1	8.01	4.98	3.04	61.0
Cu_0.03_BiTe_0.98_I_1.02_	13.6	5.66	3.90	1.76	45.1
Cu_0.05_BiTe_0.98_I_1.02_	12.1	7.24	5.24	2.00	38.2
Cu_0.05_BiTeI	14.0	4.27	2.88	1.40	48.5

Concentrations of donor (*N*_*D*_), acceptor (*N*_*A*_), and the compensation ratio (*K* = *N*_*A*_*/N*_*D*_) are calculated from *n* and *N*_*I*_.
